# Personal values, professional responsibilities and non-invasive prenatal testing: reflections of genetic counsellors in Australia

**DOI:** 10.1007/s12687-026-00913-8

**Published:** 2026-06-15

**Authors:** Chanelle Warton, Danya F. Vears

**Affiliations:** 1 School of Medicine, Deakin University, Geelong, Australia; 2 Monash Bioethics Centre, Monash University, Melbourne, Austria; 3 Murdoch Children’s Research Institute, Melbourne, Austria; 4 Department of Paediatrics, University of Melbourne, Melbourne, Austria; 5 Centre for Biomedical Ethics and Law, KU Leuven, Leuven, Belgium

## Abstract

The reproductive autonomy rationale endorses the provision of non-invasive prenatal testing (NIPT) to enable individuals to make decisions about their pregnancy in accordance with their personal values. While the personal values of healthcare professionals may promote autonomous reproductive decision-making in light of increasingly complex genetic information, they may also impinge upon reproductive autonomy through limiting patient access to care. This paper provides timely insight into genetic counsellors’ perceptions and implementation of professional responsibilities as well as their views on potential conflicts between healthcare professionals’ personal moral values and the provision of NIPT. Semi-structured interviews were conducted with 23 genetic counsellors involved in the provision of NIPT in Australia. Data was analysed using inductive content analysis. Participants expressed a shared understanding of their primary professional responsibilities, however varied in their views around operationalising this responsibility in the context of personal value conflicts. Participants reported emerging conflicts in personal values where NIPT was used to detect conditions perceived to have mild phenotypes. Instances of NIPT provision being impacted by conscientious objections to abortion among colleagues were also reported. Participants empathised with concerns of disability discrimination arising from the provision of NIPT but suggested that this could be addressed through nuances in counselling practice. Participants viewed personal values that impeded patient access to care as incompatible with reproductive autonomy. This has important implications for how reproductive autonomy is operationalised in prenatal genetic counselling practice, particularly as the complexity and quantity of genetic information returned via NIPT continues to increase.

## Background

The reproductive autonomy rationale endorses the provision of non-invasive prenatal testing (NIPT) as a means of facilitating informed decision-making (Ravitsky 2017). However, disparate conceptualisations of reproductive autonomy in turn lead to variations in views on how genetic counsellors should support informed decision-making in their clinical practice. Traditionally conceptualised, reproductive autonomy draws on individualistic notions that emphasise the pregnant person as the sole decision-maker whose choices should be free from the undue influence of others, including their healthcare provider (Jamal et al. 2020). This conceptualisation of autonomy underpins the primary approach used in current prenatal genetic counselling practice: non-directive counselling (Nuffield Council on Bioethics 2017; Garcia et al. 2008, Hodgson and Spriggs 2005; Warton et al. 2023). 

Non-directive counselling guides the professional responsibilities of genetic counsellors during the provision of NIPT. These responsibilities include providing information about the test, results and subsequent healthcare pathways free from bias or undue influence and to obtain informed consent (Jamal et al. 2020; Hodgson and Spriggs 2005). This approach typically discourages the incorporation of a healthcare professional’s personal values as a means of reducing undue influence in patient reproductive decision-making. Interpreted in this light, non-directive counselling has been critiqued for providing a negative account of professional duty (Weil 2003; Weil et al. 2006), the unattainability of true neutrality (Weil 2003) and inadequately supporting patients to navigate ethically complex decisions (Weil 2003; Weil et al. 2006, Clarke and Wallgren-Pettersson 2019). Additional interpretations of non-directive counselling have been developed (Dive and Newson 2022), including integrating non-directiveness as an element of genetic counselling practice rather than a central ethos (Weil 2003; Weil et al. 2006) or defined more broadly as promoting patient autonomy and self-directedness (Weil 2003). 

Despite the traditional emphasis on value neutrality in non-directive counselling, a healthcare professionals’ personal values may be desired by and confer benefits on patients. Patients in one study identified provider patience and attentiveness as some of the most desirable provider attributes (Schattner et al. 2004). Moreover, empathic concern among healthcare professionals has been associated with improved provider satisfaction and operational efficiency, resulting in improved patient health outcomes (Decety and Li 2025; Moudatsou et al. 2020). With the introduction and ongoing development of NIPT as a whole genome screen, the quantity of genetic information and the likelihood of returning complex results, such as variants of unknown significance or results related to multifactorial conditions, is set to increase. Allowing healthcare professionals to provide value judgements at patient behest has thus been argued to support the autonomous decision-making process in the context of complex test results (Warton et al. 2023). 

Healthcare professionals’ personal values may also be a limiting factor in patient access to NIPT, particularly, though not exclusively, when expressed as a conscientious objection. Conscientious objection refers to the refusal of a healthcare professional to participate in the provision of a lawful health service on the basis of their personal moral or religious beliefs (Fiala and Arthur 2017). While conscientious objection in the provision of NIPT is an emerging area, in prenatal screening more broadly they have primarily arisen from personal opposition to abortion (Orzechowski et al. 2021; Pawlikowski 2018) or from expressivist objections (i.e. concerns about screening perpetuating disability discrimination) (Nuffield Council on Bioethics 2017; Malek 2010; Parens and Asch 2003). Expanded uses of NIPT may provide patients with a unique opportunity to action test results in instances of uncertain phenotypes or where the implication of the result for the health of the foetus is unclear. As NIPT is increasingly integrated into healthcare systems globally, it presents a growing potential for tension between the professional responsibility to preserve reproductive autonomy and a healthcare professionals’ personal moral beliefs around termination and disability discrimination.

There is limited existing research that addresses how healthcare professionals conceptualise and operationalise their professional responsibilities and personal values in the context of NIPT (Warton and Vears 2025). This paper reports the findings of a qualitative study based on semi-structured interviews with genetic counsellors in Australia, providing insight into how genetic counsellors perceive and implement their professional responsibilities in clinical practice as well as their views on and experiences of conflict between professionals’ personal moral values and the provision of NIPT.

## Methods

Semi-structured interviews were used to understand genetic counsellors’ views and experiences of NIPT in Australia. Eligibility for this study was based on current professional registration as a genetic counsellor and current or previous involvement in the provision of NIPT in Australia. A convenience sampling approach was employed to include genetic counsellors with experience providing NIPT in Australia. Recruitment strategies included advertisements in the Human Genetics Society of Australasia newsletter, participants sharing study details with eligible colleagues and emails to participants of a previous survey who indicated their willingness to be contacted for participation in future research. A participant explanatory statement accompanied all recruitment advertisements and email invitations.

Interviews were conducted by CW via Zoom between August to September 2023 and May to June 2024. Verbal consent was provided by participants at the outset of each interview. Interviews were concluded based on the concept of information power. Information power appraises the study aim, sample specificity, use of theory, quality of dialogue and variation to determine sample size in qualitative research (Malterud et al. 2021). A semi-structured interview guide was developed and utilised to understand participants’ views and professional experiences with NIPT. Interview questions reported in this paper explored participants’ understanding of their professional obligations and their views on how healthcare professionals’ personal values could influence patient decision-making following positive NIPT results.

Inductive content analysis was used to analyse interview data. Inductive content analysis is commonly used with text-based data, such as interview transcripts, employing a close reading of texts to infer codes from the data as opposed to analysis using a pre-determined content list (Vears and Gillam 2022). Coding was a collaborative, iterative process whereby transcripts were initially coded for higher-level content categories with subcategories codes being ascertained through subsequent readings (Coulston et al. 2025). All transcripts were coded by CW. Six transcripts were independently cross-coded by DV. Codes were compared and discrepancies resolved through discussion to refine coding categories.

Ethics approval for this study was granted by the Monash University Human Research Ethics Committee (#35590).

## Results

### Participant characteristics

Interviews were conducted with 23 genetic counsellors from across Australia. Interview duration ranged from 35 to 91 min with an average of 55 min. More than half of participants had more than 10 years of practice experience (*n* = 13, 56.5%) and were employed in private health services (*n* = 14, 60.9%), which included both for-profit and non-for-profit organisations. Participants were based across all Australian states and territories with registered genetic counsellors.^1^ Participant characteristics are further described in Table 1.

**Table 1:** Participant characteristics

State	***n* (%)**
ACT	1 (4.3)
NSW	3 (13.0)
NT	0 (0)
QLD	3 (13.0)
SA	2 (8.7)
TAS	3 (13.0)
VIC	9 (39.1)
WA	2 (8.7)
Years of practice	***n* (%)**
0–5 years	5 (21.7)
6–10 years	3 (13.0)
11–15 years	6 (26.1)
16–20 years	3 (13.0)
More than 20 years	4 (17.4)
N/A	2 (8.7)
Current service type**	***n* (%)**
Private	14 (60.9%)
Public	12 (52.2%)

** Percentages equal to greater than 100 per cent as some participants held both private and public roles

The majority (*n* = 21, 91.3%) of genetic counsellors had experience with pre-test counselling for NIPT, however most (*n* = 15/21, 71.4%) described their involvement as infrequent and in limited circumstances such as for patients with a known translocation or chromosomal variation. Many public genetic counselling services in Australia provide pre-test counselling in limited circumstances as they are not resourced to provide routine counselling for population screening. Six (*n* = 6/21, 28.6%) genetic counsellors reported routinely offering and providing pre-test counselling around NIPT. All those routinely offering NIPT were employed in a private service.

All (*n* = 23/23, 100%) genetic counsellors worked with NIPT in a post-test capacity. Some noted that their post-test involvement was limited to cases where complex increased chance results were returned, such as microdeletions or sex chromosome aneuploidy, or where patients were uncertain about further diagnostic testing.

### The professional responsibilities of genetic counsellors

Genetic counsellors viewed facilitating decision-making as their principal professional responsibility across both the pre- and post-test context. Facilitating decision-making was broadly construed as combining three components: (1) providing information, (2) supporting the decision-making process and (3) navigating health systems. Information provision was understood as discussing test characteristics and possible implications of testing for patients and their family members in ways that were accessible and supported comprehension.

“*First is providing information to a patient about the genetic test result that they do have and what that means and trying to break that down so the patient can understand that result and taking the time to go through that.”* (P7).

*“Genetics is a very*,* very complex medical specialty and medical specialists don’t comprehend clinical genetics information. How could we expect someone who’s never worked a day in their life in medicine to understand half of the nonsense that we’re talking about? It’s really hard. We’re a facilitator of information…”* (P20).

Genetic counsellors believed they should take specific actions to support the decision-making process, such as ensuring options were offered and approaching a session without judgement. Some genetic counsellors viewed part of their role to be challenging patients when their values and actions appeared discordant.

*“I think a lot of genetic counsellors get very nervous about challenging anybody’s decision-making but again it’s the process to me. We have a responsibility sometimes to sit uncomfortably in the session taking those challenges where we may not be their favourite person at the end of the conversation but they will have thought about things they might not have thought about.”* (P13).

*“I would get a bit more involved when people seem to be a bit discordant*,* when they express values or wants in one direction that seem to be making the opposite decision that would fit with that.”* (P4).

The final component of facilitating decision-making was navigating healthcare systems, where genetic counsellors expressed a responsibility to advocate for their patients, liaise with other healthcare professionals and connect patients to relevant health services.

*“That includes some advocacy so also doing a lot of keeping different doctors or the other clinicians in the loop and making sure that they have the information in a way that makes sense to them and having and being aware of some of the psychological stuff around making decisions.”* (P16).

*“… also being able to know which services to link them in with and how you fit into the picture with the other health professionals that are involved in their care.”* (P18).

In support of facilitating decision-making, genetic counsellors often reported an obligation to pursue continued professional development. This included staying informed about test developments, revising approaches to genetic counselling practice and engaging with perspectives of diverse stakeholders.

*“… a responsibility to have accurate information*,* so that means being up to date with information and making sure that you review information and that you’ve got as much relevant information as you can.”* (P16).

Participants perceived all genetic counsellors as having the same core responsibilities. These were often reported as arising from professional society guidelines, specifically the Human Genetics Society of Australasia (HGSA) Scope of Practice and Code of Ethics, and registration requirements.

*“We have core competencies that are provided through the Human Genetics Society of Australasian and the Australasian Society of genetic counsellors. We have a code of ethics which most people*,* when they’re new to the profession*,* aren’t really that familiar with… You have to learn how to apply those competencies and those ethical frameworks to what you’re doing.”* (P13).

*“The HGSA has clear requirements and guidelines around what genetic counsellors can or should do or how much workload they should have et cetera. At the core*,* I think our responsibility is the same or very similar across different areas of genetics…”* (P20).

However, many noted that the operationalisation of these responsibilities was dependent on individual context such as management and the healthcare professionals and services they worked with.

*“*[A genetic counsellor’s professional responsibilities] *can very much depend on their place of work*,* what the expectation is of them within their place of work*,* what their experience is to be able to do the job.”* (P22).

*“Knowing how* [professional responsibilities] *apply to your role*,* where you’ve reached the edge of your scope– What you do with those things will look different depending on where you practice and what your core roles are*,* but they’re essentially the exact same responsibilities…”* (P13).

This aligned with views that a responsibility to provide counselling for NIPT was context specific. Perceptions of which contextual aspects determined this responsibility varied, with some suggesting the responsibility was determined by individual workplaces while others pointed to general areas of practice or offering the test as determining factors.

*“I do think that it’s under our professional opportunities. We are supposed to provide information about the options of genetic screening and diagnostic testing in a reproductive setting… Resulting and high-risk results*,* absolutely*,* we are best placed to do that and it definitely falls within our professional guidelines and our professional duty to be able to do that…”* (P19).

*“The services that do offer it definitely have an obligation to know what it does and how good it is and to be able to communicate that to the clients.”* (P21).

### Experiences of managing personal values in current practice

Some genetic counsellors reported feelings of discomfort about a patient’s decisions around NIPT. This discomfort largely arose where patients chose to terminate pregnancies affected by conditions that genetic counsellors perceived to be mild or in instances of non-medical sex selection.

*“When people are making decisions about terminations for sex chromosome abnormalities. I’m completely patient-focused*,* and completely supportive of their decisions… It makes me a little bit sad when people decide to stop a pregnancy.”* (P3).

*“Making a decision to end a pregnancy based on Triple X syndrome when the likely phenotype is very*,* very mild. That can be challenging. But*,* at the end of the day*,* I have to support and help a patient through making those difficult choices. It frightens me a little bit sometimes that we’re striving for perfection in our pregnancies and that a slightly*,* maybe 5 to 10 lowered points in IQ– that could happen to anybody and*,* to me personally*,* why would you end a pregnancy based on that?”* (P6).

Several genetic counsellors additionally reflected their experiences balancing concerns of perpetuating disability discrimination and their professional role to provide information and support decision-making about prenatal screening for disability:

*“It’s something that I think about all the time…* [I am involved with disability communities because] *I can’t practice the way that I want to practice if I don’t have an investment in the people who exist with genetic conditions*,* knowing that you have to have a full conversation with people… That’s one of the real failures of pre-test counselling is that people aren’t having a conversation about why they’re doing the test before they’re doing it and a whole bunch of assumptions are made about what they will do with that information once they get it…”* (P13).

*“I’m going to disclose; I personally struggle with a lot of this stuff…. It’s that really fine line that needs to be walked between what is the purpose of the test and if the purpose is about providing people with information to make good decisions*,* then that’s fine. But if the purpose is about saying you don’t have a child with this condition*,* then that’s not okay.”* (P14).

All genetic counsellors who reported an experience of personal discomfort emphasised that prenatal screening decisions were ultimately that of the patient and that their role was to support the decision-making process. Most genetic counsellors reported that their experiences of personal discomfort did not affect their professional recommendations. While no genetic counsellors reported needing or wanting to remove themselves from a case, a small number suggested they would consider doing so if they felt unable to adequately facilitate patient decision-making.

*“If I felt that it went against a moral compass of mine*,* I wouldn’t do it. I’d step away… I wouldn’t offer it through our service if it was something I felt extremely uncomfortable about and I don’t think my boss would let that happen either.”* (P6).

Several genetic counsellors highlighted the unique training and support available, particularly supervision, to them when providing care to patients who made decisions not aligned with the GC’s personal values.

*“A big part of genetic counselling practice is to have supervision and to be aware of your own biases*,* as much as you can be*,* or what’s making you uncomfortable*,* unpacking some of that. But yeah*,* certainly I feel maybe there’s been situations sometimes where I think ‘I’m not sure if that was the best decision’.”* (P9).

*“This is one of the things that*,* as genetic counsellors*,* we’re trained to think through and to work through in our training and to acknowledge that yeah*,* there are decisions that people will make that you might that maybe you don’t necessarily agree with*,* or you’re not comfortable with*,* or have to seek some support and supervision around.”* (P14).

Beyond their personal values, a number of genetic counsellors raised real examples of healthcare professionals, some of whom held genetics training, who held personal beliefs against abortion and explained how this influenced patient care related to NIPT in current practice.

*“I’ve seen some of this*,* so I don’t think they should be involved… I’ve worked with somebody who is very religious and doesn’t believe in termination. I don’t think that they’ve always done the right thing in terms of directing the patient* [following NIPT] *and doing procedures that the patient has requested*,* because of their own beliefs.”* (P10).

*“I had a patient once who actually… had a trisomy 21 result*,* which was a really complex chromosomal rearrangement and she got it at 20 weeks…* [Colleague] *was happy to facilitate her NIPT but once she got a diagnosis and it wasn’t going to be a straightforward Down syndrome result*,* it was going to be a more complex result…* [they] *said ‘I’m not prepared to arrange a pregnancy termination for you.’… She had to do it all alone because she didn’t have any support but she knew she was going into that and she then found out that her practitioner wouldn’t support her either and she got ping-ponged around multiple hospitals where nobody really wanted to take responsibility or ownership for her.”* (P11).

“[Colleague] *has a very strong Catholic faith.* [Colleague] *does not believe in termination of pregnancy*,* and so*,* if* [they] *see an abnormality on ultrasound*, [they] *will not say to the patient*,* ‘You should do an amnio or you should do a CVS’ and* [they] *certainly don’t perform KCL injections or medication to bring on… But I’ve worked in practices that do and* [colleague] *doesn’t believe in that*,* but* [they] *employ genetic counsellors that do… so that we can discuss those things so that* [they don’t] *have to.”* (P6).

Genetic counsellors additionally distinguished between their experiences of objections held by individual healthcare professionals and religious bans placed on termination of pregnancy at an institutional level. Participants reflected on their experiences of institutional refusals to provide termination of pregnancy, particularly as creating challenges in fulfilling their professional role of connecting patients with subsequent services. Institutional refusals to termination of pregnancy were reported as arising from both public and private hospitals that provided other specialist maternal health services, including prenatal screening.

*“The… Catholic hospital won’t offer terminations of pregnancy and I find that extremely frustrating because they leave people without simple options… You can work around an individual if you have the information but it’s very hard to work around a service…”* (P16).

### Conflict between healthcare professionals’ personal values and professional responsibilities

Genetic counsellors were additionally asked for their views on hypothetical scenarios in which healthcare professionals more broadly were opposed to providing prenatal screening based on their personal beliefs. Moral opposition to abortion and concerns about disability discrimination were provided to participants as examples of personal beliefs that hypothetically could impact a healthcare professional’s provision of NIPT. Many genetic counsellors felt strongly that healthcare professionals were obligated to provide prenatal screening and that professional duties could not be selectively fulfilled on this basis. Suggested management strategies included for healthcare professionals to transition out of areas of practice where they were likely to encounter patients requiring prenatal screening.

*“You have to be really aware of that intersection and check yourself constantly to make sure that you’re able to provide good care to that person and if you genuinely don’t think you can*,* you need to excuse yourself and not practice in an area where that’s going to be something that you encounter frequently.”* (P13).

*“I think people might have found themselves in the wrong profession.”* (P3).

Some genetic counsellors suggested that healthcare professionals should put aside their personal beliefs to fulfil their professional responsibility of offering prenatal screening. However, others considered greater benefits for patients to not be provided prenatal screening by healthcare professionals who opposed it.

*“If you definitely have a moral problem with it*,* then obviously*,* you’re not going to be the best person to provide that patient care. And I feel like if they were under obligation to provide consent or information on it*,* I don’t know if I would trust that information that’s coming from somebody who’s against prenatal screening.”* (P2).

*“… if they have a conscientious objection to doing it*,* then they shouldn’t do it. For their sake and the patient’s sake.”* (P10).

No genetic counsellors supported an absolute right of healthcare professionals to choose not to provide prenatal screening based on their personal beliefs. Where healthcare professionals refused to be involved in prenatal screening provision, genetic counsellors emphasised the need to reduce subsequent inequity through ensuring patients were aware of the full range of care available and facilitating referrals.

*“I see* [referrals] *as being pretty integral to the follow-up of a result that they’ve received*,* so I think there does need to be that follow-up. That’s not to say that it’s easy to navigate the system*,* so I do certainly sympathise for doctors that it’s often tricky to link their patients in with care*,* it might be inadequate care*,* so it’s often not an ideal process*,* and it’s hard*,* often for GPs*,* to know where to turn to for that support as well. So I do think it’s a bit of a challenge*,* but I do think the responsibility does lie with the requesting healthcare provider.”* (P1).

*“*[Healthcare professionals] *might argue that referring on is facilitating the same action*,* and so they may have an objection against that*,* but I don’t know – I can’t understand why somebody would work in an area like prenatal care and conscientiously object to a specific aspect of that care*,* but then refuse to support the client in a decision that they want to make. So I think they do have an obligation to refer.”* (P5).

Genetic counsellors raised further concerns about inequity where healthcare professionals refused to be involved in prenatal screening provision, particularly if providers were based in remote or rural settings.

*“It might be a small town where there’s only a small number of GPs or doctors available so it is tricky and some patients are going to lose out because of that. That’s the inequality of healthcare anywhere.”* (P17).

*“If you’re in a well-equipped service where it’s easy enough to hand over to somebody else*,* then I suppose that’s probably better for everybody. But if you took things to its logical extreme and you are the only doctor in a small town and you work for public service then I think you do have a responsibility to let people know what’s available.”* (P4).

Genetic counsellors additionally raised several considerations related to the values driving a healthcare professional’s hypothetical opposition to some aspect of NIPT provision. Where healthcare professionals’ opposition was motivated by their views on abortion, all but one genetic counsellor felt there remained an obligation to facilitate abortion or, at least, provide referrals. One genetic counsellor suggested that healthcare professionals had an obligation to *offer* screening but were not obligated to facilitate or refer for termination following a positive result:^2^

*“... that you have a right to screening*,* I think most doctors would agree with that. The problem is*,* if that screening comes up high risk*,* ‘I will not offer you a termination or provide you a referral for a termination’. That is very different. I would believe that a personal belief related to the provision of a termination is absolutely within the right of a health professional to make that decision for themselves. But the screening prior to that*,* it’s mandated. You have to offer screening.”* (P19).

Genetic counsellors expressed conflicting views about whether a healthcare professional’s personal views on abortion should be disclosed to patients.

*“I would hope that the patient would be protected from knowing that.”* (P10)

*“I feel like the healthcare professional needs to be upfront in their views and opinions and say*,* ‘There is this testing available*,* I’m not comfortable in counselling*,* offering or doing that.’ I know it’s not always practical.”* (P17).

Conflicting views were also expressed about whether there was a sufficient association between prenatal screening and abortion to justify a refusal to provide NIPT.

*“I think it’s very different giving women the capacity to find out about their baby before they’re born as opposed to facilitating a termination… So I actually feel quite strongly that they should be providing that test*,* that service*,* if that’s part of their role…”* (P12).

*“I guess, at the end of the day you are providing screening to understand whether there are any significant health problems that would influence the parents to choose to stop the pregnancy*,* that’s the most common reason. It is like the first step on that staircase up to potential termination*,* so I could understand why they would choose to not do prenatal screening if they didn’t want to get to the top of the stairs.”* (P5).

Genetic counsellors were also asked to consider refusals to be involved in the provision of prenatal screening arising from concerns about discrimination against people with disabilities. While some genetic counsellors noted disability concerns would be reasonable grounds to refer patients to another healthcare professional, most genetic counsellors emphasised the need to support individual reproductive decision-making and offered alternative strategies for providing balanced prenatal genetic counselling.

*“Education helps all of us at the end of the day. We can all be educated on things we don’t understand. I understand the perception that prenatal testing devalues people with disability… It is the responsibility of the disability community to educate because they’re the best placed people to educate about what living with the disability looks like and*,* by the same token*,* we still have to be mindful of individual’s rights.”* (P22).

“[Healthcare professionals] *should address those feelings elsewhere. Withholding the ability to have a test like that isn’t the appropriate way forward when we’re thinking about making our society more open and understanding and tolerant of people that are different from one another. It’s theoretically just still not up to them. Perhaps they would be better suited in advocacy or another role.”* (P4).

## Discussion

According to the our knowledge, this is the first study to examine the relationship between genetic counsellors’ personal values and their professional responsibility to facilitate reproductive autonomy in the provision of NIPT. While some participants experienced personal discomfort in response to the reproductive decisions of some patients, they did not personally report experiences of moral distress or conscientious objection. Conflicts between genetic counsellors’ personal values and professional responsibilities related to NIPT has received limited attention and is often reported as an incidental research finding. For example, Belgian genetic counsellors in a 2024 study noted their right to conscientiously object to sex-selective abortion arising from NIPT: they reported making normative value judgements while providing prenatal counselling and reporting withholding or delaying information provision (Claesen-Bengtson et al. 2024). However, further literature highlights conscientious objection among healthcare professionals to prenatal testing more broadly (Orzechowski et al. 2021; Haidar et al. 2020). Among participants in this study who reported experiences involving colleagues with conscientious objections, all viewed the personal values of healthcare professionals as an insufficient justification for limiting or delaying patient access to care.

Genetic counsellors expressed a shared understanding of their overarching responsibility to support informed decision-making. Facilitating decision-making was considered to comprise information provision, providing support during the decisional process and navigating healthcare systems on behalf of patients. This aligned with professional responsibilities and practice competency standards and scope of practice for genetic counsellors outlined by the HGSA (2022a, b). While genetic counsellors expressed a shared higher-level understanding of their professional responsibilities, views on how responsibilities were operationalised at an individual level varied. Some participants identified challenging discordant patient decisions as part of their role in supporting informed decision-making. Challenging patients where patient values and decisions appear discordant reflected utilisation of a broader understanding of non-directiveness that extends genetic counsellors’ roles beyond that of a neutral information provider (Weil 2003). 

Conflicts between the personal moral values of healthcare professionals and the provision of NIPT is an ethical and clinical practice issue. Genetic counsellors in this study reported moral discomfort where NIPT was used to detect conditions with mild phenotypes, particularly sex chromosome aneuploidies, or foetal sex for non-medical sex selective termination. This concern is reflected in theoretical bioethics literature that considers the parameters and utility of the seriousness of a condition as a threshold for NIPT use. The ‘seriousness’ of a genetic condition lacks conceptual clarity (Kleiderman et al. 2025). While many definitions focus on perceived objective clinical indicators (Kleiderman et al. 2025; Bayefsky and Berkman 2021), others incorporate lived experience and socioeconomic and cultural factors (Kleiderman et al. 2025; Kleiderman et al. 2022). Beyond challenges with conceptual clarity, the utility of seriousness as a threshold for testing use has also been questioned. Some argue that a seriousness threshold would be arbitrarily determined and such arbitrary restrictions would impinge upon autonomous reproductive decision-making (Taylor-Sands et al. 2025). Others argue that a seriousness threshold ensures patients are presented with information material to making meaningful reproductive choices, as opposed to all information, avoids a slippery slope towards testing and termination for non-medical traits and protects the interests of the potential future person (Nuffield Council on Bioethics 2017, Dondorp et al. 2015). 

As NIPT expands to detect further conditions with varying degrees of severity, moral conflict among healthcare professionals is likely to become more prevalent. While genetic counsellors in this study experienced moral discomfort in response to some patient decisions, they did not report moral distress themselves. There may be unique factors among the profession that protect genetic counsellors from moral distress, such as access to supervision as a means of understanding ethical resolution where values conflict. In this study, most genetic counsellors who experienced personal moral discomfort emphasised utilising supervision as a means of managing personal discomfort. Understanding the primary goal of NIPT as facilitating reproductive autonomy, as opposed to as designed to reduce the population incidence of certain conditions (Ravitsky 2017), may additionally act as a protective factor against moral distress. NIPT is being increasingly provided by non-genetics professionals who may not have access to similarly protective factors. Additional support resources may be required for genetic counsellors where NIPT is increasingly used for perceived milder conditions. Education and support resources are needed for non-genetics professionals to aid in the management of personal moral discomfort or distress during the provision of NIPT for diverse conditions.

While genetic counsellors in this study did not restrict access to termination of pregnancy following NIPT, a small number of examples of other healthcare professionals restricting access to abortion in the context of NIPT were identified. Some genetic counsellors reported the personal moral views of a healthcare professional influencing post-test recommendations and limiting patient access to subsequent care. This reflects existing clinical practice issues relating to the provision of prenatal screening more broadly, where conscientious objections to abortion are the primary cause of objections to involvement (Orzechowski et al. 2021; Pawlikowski 2018; Slokenberga et al. 2019). Genetic counsellors in this study suggested that the personal moral objections of healthcare professionals should not supersede patient access to NIPT and subsequent care, including pregnancy termination. This aligns with some ethics discourse that recognises a need to protect the moral integrity of healthcare providers while simultaneously protecting and facilitating the right of patients to care (Brock 2008; Magelssen 2012). The impact of healthcare professionals’ conscientious objections to abortion are likely to be exacerbated given the wider range of conditions detectable via NIPT and increasing uptake of the test.

Concerns among healthcare professionals of increasing discrimination and stigmatisation of people with disabilities arising specifically from NIPT use is an emerging issue in academic literature (Nuffield Council on Bioethics 2017; Warton and Vears 2025; Haidar et al. 2020). Many genetic counsellors empathised with the expressivist objection and reported difficulties balancing discrimination concerns with NIPT provision in their clinical practice. However, all emphasised a continued commitment to supporting autonomous choice in reproductive decision-making and that concerns about disability discrimination could be compatible with patient choice if incorporated into practice in nuanced way. This aligns calls for more nuanced discussions and increased access to balanced resources for prospective parents of a child with a disability (Nuffield Council on Bioethics 2017; Hippman et al. 2012; Schendel et al. 2017). Expressivist objections among healthcare professionals may increase as NIPT is used to screen for additional conditions (Nuffield Council on Bioethics 2017). Care should be taken to ensure patients using NIPT have access to balanced information about a given disability where NIPT is integrated into health services.

### Strengths and limitations

This study, to our knowledge, is the first to examine the relationship between genetic counsellors’ personal views and values and the professional responsibility to facilitate autonomous decision-making around NIPT. A strength of this study lies in the qualitative semi-structured interview design that supported rich, in-depth examination of genetic counsellors’ experiences and perspectives. While convenience sampling was useful for recruiting busy participants and exploring emerging ethical and clinical practice themes, there are limitations with this sampling approach. Convenience sampling may introduce selection bias and, while a high level of diversity across professional characteristics was reported, participants may not be wholly representative of the genetic counselling profession. This study also explored genetic counsellors’ views on hypothetical scenarios where a healthcare professional’s personal views about NIPT conflicted with their professional responsibilities. As this is an emerging area of research and clinical practice, participants may not have previously reflected on these ideas and their views may evolve alongside related clinical experiences. Future areas of research may explore personal-professional value conflicts related to NIPT among other health professions or further exploration of how genetic counsellors navigate concerns about potential disability discrimination in prenatal screening.

## Conclusion

As the complexity and quantity of genetic information returned via NIPT grows and the test is increasingly integrated into healthcare systems, there is a need to consider the role of genetic counsellors’ personal values in prenatal genetic counselling. Understanding how genetic counsellors perceive and manage conflicts between professional responsibilities and personal values is crucial in facilitating pregnant persons’ reproductive autonomy. Genetic counsellors expressed a shared view that personal values that impeded patient access to care were incompatible with reproductive autonomy. However, many genetic counsellors questioned whether value neutrality was achievable or useful in practice and reported directivity in the decisional process. While genetic counsellors experienced some moral discomfort about patient decisions, this did not extend to moral distress and did not impede patient access to care. Additional resources and supports may be required for non-genetics professionals as the scope and uptake of NIPT increases to protect them from moral distress. Genetic counsellors hold unique expertise in supporting reproductive autonomy in prenatal genetic counselling and further research is required to assess variations in clinical practice where provided by non-genetics professionals.

## Data Availability

Data generated as part of this study are not publicly available due to consideration of the risk of identifiability of participants.

## Footnotes

1 Participants were based across 7 of 8 Australian states and territories. There are currently no genetic counsellors who hold practice registration in the Northern Territory. No participants were recruited from the Northern Territory.

2 In Australia, medical practitioners who hold conscientious objections to termination of pregnancy are legally obligated to facilitate referrals.
